# Case Report: Hypereosinophilic syndrome misdiagnosed as atopic dermatitis due to refractory pruritic rash masking peripheral neuropathy

**DOI:** 10.3389/fmed.2026.1794649

**Published:** 2026-03-25

**Authors:** Huan Li, Xiaoping Yang, Caixia Zhang, Wanling Zhu, Honglei Jia

**Affiliations:** 1 Department of Neurology, 970th Hospital of PLA Joint Logistic Support Force, Yantai, China; 2 Department of Laboratory, 970th Hospital of PLA Joint Logistic Support Force, Yantai, China; 3 Department of Endocrinology, 970th Hospital of PLA Joint Logistic Support Force, Yantai, China; 4 Yantai Fourth Retired Cadres' Recuperation Institute of Shandong Military Region, Yantai, China

**Keywords:** anti–IL-5 therapy, atopic dermatitis, hypereosinophilic syndrome, peripheral neuropathy, refractory pruritus, sensory masking

## Abstract

**Background:**

Hypereosinophilic syndrome (HES) is a rare and heterogeneous disorder characterized by persistent eosinophilia and multi-organ involvement. Cutaneous manifestations are common, whereas peripheral neuropathy may be underrecognized, particularly when dermatologic symptoms predominate.

**Case presentation:**

We report the case of a 49-year-old man with a 10-year history of recurrent pruritic papules initially managed as atopic dermatitis. Laboratory testing revealed marked eosinophilia (absolute eosinophil count: 5.29 × 10^9^/L) and significantly elevated total immunoglobulin E (IgE) (>6,000 IU/mL; reference range: 0–100 IU/mL). One week after initiating upadacitinib for refractory pruritus, the patient’s itch severity decreased substantially, after which he more clearly perceived distal numbness and gait instability. Nerve conduction studies demonstrated a symmetric sensory-predominant axonal polyneuropathy. Skin biopsy showed eosinophilic inflammatory infiltration without evidence of vasculitis. Quantitative intraepidermal nerve fiber density (IENFD) was within the age-adjusted reference range, consistent with preserved small-fiber integrity. After comprehensive exclusion of secondary and clonal causes of eosinophilia, a diagnosis of idiopathic HES with cutaneous and peripheral nervous system involvement was established. Treatment was adjusted to mepolizumab (300 mg subcutaneously every 4 weeks), followed by clinical improvement in neuropathic symptoms and a reduction in eosinophil levels.

**Conclusion:**

This case highlights the importance of maintaining a high index of suspicion for systemic eosinophilic disorders in patients with chronic refractory pruritus and marked eosinophilia. Severe pruritus may obscure or delay recognition of concurrent neurological symptoms; therefore, early electrophysiological evaluation should be considered when systemic involvement is suspected. Targeted anti-IL-5 therapy represents an effective treatment strategy in selected cases of HES.

## Introduction

Hypereosinophilic syndrome (HES) is a heterogeneous group of disorders characterized by persistent and marked peripheral blood eosinophilia (typically ≥ 1.5 × 10^9^/L) associated with organ damage and/or functional impairment, after the exclusion of other secondary causes such as clonal or reactive eosinophilia (1, 2). The clinical manifestations of HES are highly diverse and may involve multiple organ systems, including the skin, lungs, gastrointestinal tract, heart, and nervous system. The skin is one of the most frequently affected organs in HES, with approximately 37–69% of patients developing cutaneous manifestations. These presentations are heterogeneous and include eczematous dermatitis, erythroderma, and urticaria. Consequently, HES is frequently misdiagnosed as common inflammatory skin diseases, such as atopic dermatitis (AD), leading to delayed or incorrect diagnoses (3, 4).

Neurological involvement occurs in a substantial proportion of patients with HES and may manifest as peripheral neuropathy and/or central nervous system symptoms. Among these, HES-associated peripheral neuropathy typically presents as a symmetric, axonal polyneuropathy on both clinical and electrophysiological evaluation. The underlying mechanisms are believed to be primarily related to eosinophilic infiltration, the release of cytotoxic granule proteins (such as major basic protein), or ischemic injury caused by the obstruction of small endoneurial vessels, rather than classic necrotizing vasculitis (5). These features distinguish HES-related neuropathy from that observed in eosinophilic granulomatosis with polyangiitis (EGPA), another eosinophilic disorder frequently associated with neuropathic involvement. EGPA is commonly accompanied by asthma and a prodromal allergic history, with approximately 30–40% of patients being positive for anti-neutrophil cytoplasmic antibodies (ANCAs). Neuropathy in EGPA more typically manifests as mononeuritis multiplex and is pathologically characterized by vasculitic changes (6, 7). Nevertheless, differentiating idiopathic HES from ANCA-negative EGPA in patients without asthma remains a major clinical challenge, as the two conditions share substantial overlap in clinical manifestations (5).

In this report, we describe the case of a patient with HES who presented with prominent refractory pruritus over a 10-year disease course, during which manifestations of peripheral neuropathy were not clearly recognized. Through a detailed analysis of the patient’s clinical presentation, electrophysiological findings, pathological features, and therapeutic responses, together with a review of the relevant literature, this case report aims to elucidate the key differential diagnostic features distinguishing HES from AD and ANCA-negative EGPA. We further explore the potential mechanisms and clinical implications of severe pruritus, potentially delaying recognition of neurological symptoms; highlight the significance of the dissociation between neuropathological findings (PGP9.5 immunostaining) and electrophysiological abnormalities; and analyze the role of targeted therapies—particularly anti–interleukin-5 treatment—in controlling disease activity and stabilizing neurological involvement in HES. We hope that this case will provide practical insights for clinicians in the evaluation and management of patients with chronic pruritus accompanied by eosinophilia.

## Clinical information

A 62-year-old man was admitted to our hospital with a 10-year history of recurrent generalized brownish papules accompanied by severe pruritus, which had worsened and was associated with gait instability for 1 week. Ten years earlier, during winter, the patient developed dense brownish maculopapular eruptions on both lower legs without an identifiable trigger, accompanied by intractable pruritus. He was initially evaluated at a local hospital and diagnosed with atopic dermatitis or allergic dermatitis. Treatment with systemic corticosteroids resulted in incomplete symptom relief. Over the following years, he sought care at multiple medical institutions and received various systemic treatments, including *Tripterygium wilfordii* glycosides, betamethasone, thalidomide, cyclophosphamide compound tablets, levocetirizine hydrochloride, and doxepin, along with topical corticosteroids such as halcinonide, fluocinolone acetonide, mometasone furoate, and halometasone creams. His symptoms fluctuated but were never fully resolved.

One week prior to admission, after discontinuation of medications, the patient experienced a marked exacerbation of pruritus accompanied by gait unsteadiness, described as a swaying sensation while walking, prompting referral to our institution. His medical history was unremarkable for asthma, nasal polyps, and food or drug allergies, and there was no family history of hereditary disease.

On physical examination, the patient was alert but appeared fatigued. Higher cortical functions, including memory, calculation, and comprehension, were grossly intact. Patchy erythematous papules were noted on the trunk and extremities. Cranial nerve examination was normal. Muscle strength was graded 5 in all four limbs, with normal muscle tone. Finger-to-nose and heel-to-shin tests were performed accurately. Romberg’s test was negative with both eyes open and closed. Pain and temperature sensations were symmetric in the face and extremities. Deep tendon reflexes, including biceps, triceps, patellar, and Achilles reflexes, were normal. Pathological reflexes (Babinski and Chaddock signs) were absent. There were no signs of meningeal irritation.

Laboratory investigations revealed normal liver, renal, cardiac function, and electrolyte levels. Immunological testing showed markedly elevated serum immunoglobulin E (>6,000 IU/mL; reference range: 0–100 IU/mL), while immunoglobulin A, D, and G levels were within normal ranges. Complete blood count demonstrated significant eosinophilia, with an absolute eosinophil count of 5.29 × 10^9^/L (56.1%) and macrocytosis. D-dimer was elevated to 7.41 μg/mL. Vasculitis screening, including c-ANCAs, p-ANCAs, proteinase 3 (PR3), and myeloperoxidase (MPO), was negative. Antinuclear antibody profile, thyroid function tests, infectious disease screening, and urinalysis were all unremarkable.

Brain magnetic resonance imaging (MRI) and magnetic resonance angiography (MRA) showed no abnormalities. Chest computed tomography revealed small bilateral pulmonary nodules and localized pleural thickening. Transthoracic echocardiography demonstrated impaired left ventricular diastolic function. Abdominal ultrasonography showed no abnormalities in the liver, gallbladder, pancreas, spleen, or kidneys. The initial working diagnosis was gait instability of unclear etiology.

**Figure 1:**
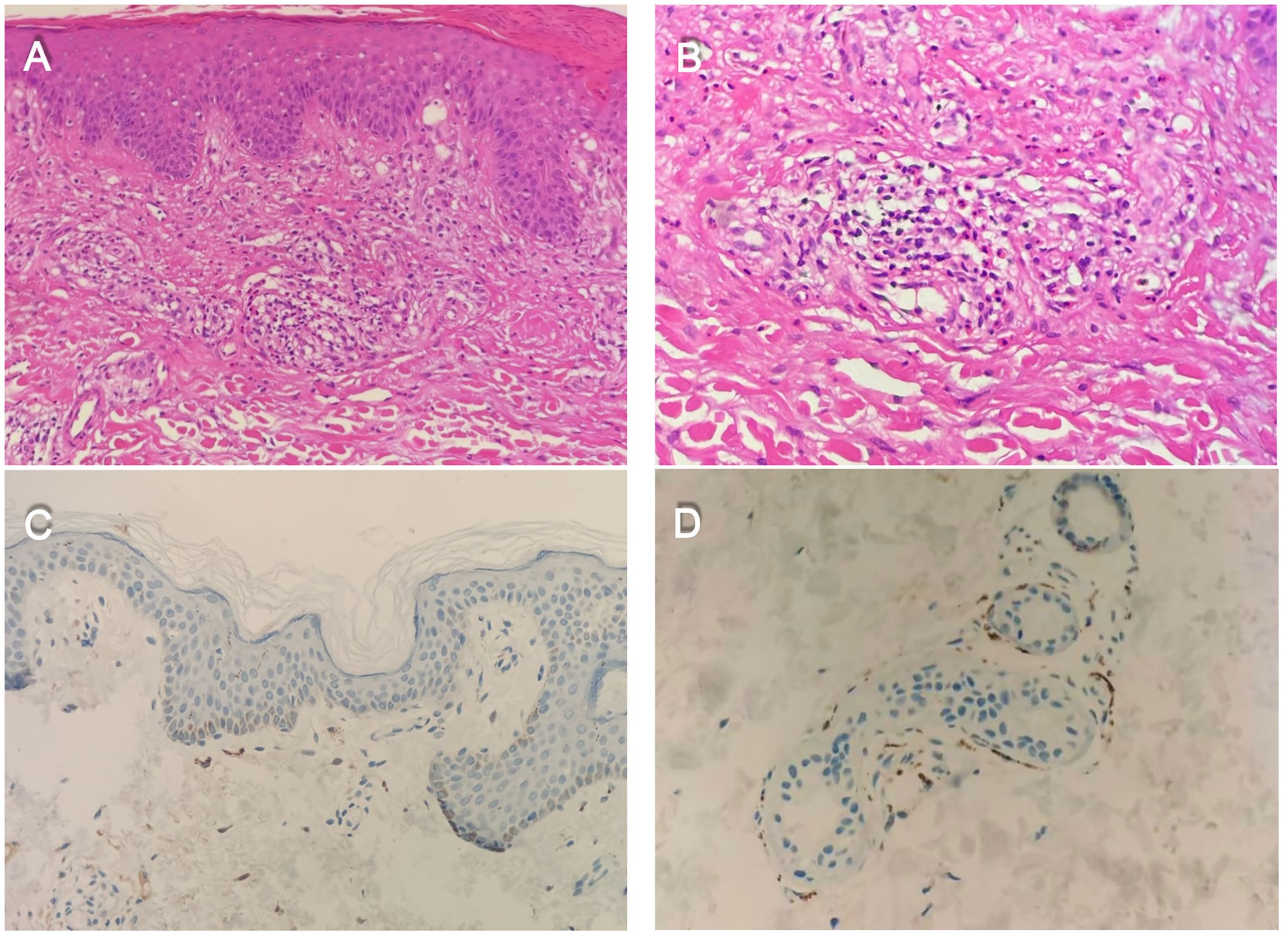
Histopathological findings of a lower-leg skin biopsy from a patient with hypereosinophilic syndrome. **(A)** Perivascular inflammatory cell infiltration is present in the superficial dermis, predominantly composed of small lymphocytes, with scattered eosinophils (HE x20). **(B)** Scattered eosinophils are observed in the superficial dermis, with preserved vascular lumina and no evidence of fibrinoid necrosis, thrombosis, or leukocytoclastic nuclear debris (HE x40). **(C)** Nerve fibers are preserved in the epidermis and superficial dermis, without a reduction in intraepidermal nerve fiber density (PGP9.5 ×20). **(D)** Nerve fibers surrounding sweat glands are preserved, without evidence of nerve fiber loss (PGP9.5 ×40).

Following dermatology consultation, an open skin biopsy was performed from the distal lower leg (approximately 10 cm proximal to the lateral malleolus). The specimen was fixed in 4% paraformaldehyde, cryoprotected, and sectioned at 50 μm thickness. Histopathological examination showed an intact epidermis with mild acanthosis. Mild perivascular inflammatory cell infiltration was observed in the superficial dermis, predominantly composed of small lymphocytes with scattered eosinophils. Vascular lumina were preserved, with no evidence of fibrinoid necrosis, thrombosis, or leukocytoclastic debris. The dermal collagen bundles appeared loose with mild interstitial edema. Immunohistochemical staining using anti-PGP9.5 antibody was performed to assess intraepidermal nerve fiber density (IENFD). Quantitative analysis demonstrated an IENFD of 8 fibers/mm, within the laboratory age-adjusted reference range (≥ 4 fibers/mm). These findings indicate preserved small intraepidermal nerve fibers (Figure 1).

Based on these findings, the dermatology team considered a diagnosis of severe atopic dermatitis and initiated treatment with upadacitinib extended-release tablets (15 mg orally once daily), along with adjunctive anti-inflammatory and antipruritic therapies. Given the markedly elevated baseline D-dimer level (7.41 μg/mL) and the known class warnings regarding thromboembolic risk associated with Janus kinase inhibitors, a focused thrombotic risk assessment was performed prior to therapy initiation. The patient had no history of venous thromboembolism, malignancy, recent immobilization, or cardiovascular risk factors. Physical examination revealed no signs suggestive of deep vein thrombosis, and no clinical evidence of active thrombosis was identified. In the absence of symptoms or imaging findings indicative of thromboembolic disease, anticoagulation prophylaxis was not initiated. During treatment and subsequent follow-up, no thromboembolic events or other major adverse events were observed. Hematology consultation was obtained, and molecular testing for the FIP1L1–PDGFRA fusion gene was negative.

After 1 week of treatment with upadacitinib, the patient’s pruritic papules improved markedly. Pruritus severity was retrospectively quantified using an 11-point numeric rating scale (Itch-NRS) at the time of admission. The patient reported an average itch intensity of 9/10 prior to treatment, associated with significant sleep disturbance (awakening 3–4 times nightly). One week after the initiation of therapy, itch intensity decreased to approximately 2–3/10, with substantial improvement in sleep quality. During this period of reduced pruritus, gait instability persisted, and the patient began to clearly perceive and report numbness and distending pain in both lower extremities, described as a “cotton-walking” sensation on the soles. Neurophysiological evaluation was performed using standard nerve conduction study (NCS) protocols. Sensory nerve conduction studies included the bilateral median, ulnar, radial, sural, and superficial peroneal nerves. Sensory nerve action potentials (SNAPs) of the bilateral sural and superficial peroneal nerves were absent. Median and radial SNAP amplitudes were reduced with slowed conduction velocities. Motor nerve conduction studies were performed on the bilateral median, ulnar, peroneal, and tibial nerves. Reduced compound muscle action potential (CMAP) amplitudes were observed in the ulnar nerves bilaterally, with focal slowing across the left elbow segment. Distal latencies and F-wave latencies were within normal limits. Needle electromyography (EMG) was performed on selected muscles of the right upper and lower limbs. No spontaneous activity, including fibrillation potentials or positive sharp waves, was observed. Motor unit potentials were within normal limits, and no active denervation changes were identified. The overall electrophysiological pattern was consistent with length-dependent, sensory-predominant axonal polyneuropathy. Electrophysiological findings are summarized in Table 1. Detailed motor and sensory nerve conduction data are provided in Supplementary Tables S1–S3.

**Table 1:** Summary of electrophysiological findings.

**Examination**	**Nerves/Regions**	**Key Findings**	**Interpretation**
Motor NCS	Bilateral ulnar nerves	Reduced CMAP amplitudes; focal slowing across the elbow on the left	Suggests focal ulnar neuropathy
	Other motor nerves	Distal latencies and conduction velocities were preserved	No generalized motor demyelination
Sensory NCS	Sural, superficial peroneal (bilateral)	SNAP absent	Length-dependent sensory axonal neuropathy
	Median, radial nerves	Reduced SNAP amplitudes	Symmetric sensory involvement
F-wave	Median, tibial	Present with normal latencies	No proximal conduction abnormality
Needle EMG	Upper and lower limbs	No spontaneous activity; normal motor unit action potentials (MUAPs)	No active or chronic denervation

Following the confirmation of symmetric sensory-predominant axonal neuropathy on electrophysiological examination, a comprehensive diagnostic reassessment of persistent marked eosinophilia (absolute eosinophil count: 5.29 × 10^9^/L) was undertaken to establish the disease classification. Reactive causes of eosinophilia were systematically evaluated and excluded. There was no history of asthma, allergic rhinitis, or drug exposure temporarily associated with eosinophilia. Infectious screening, including parasitic evaluation, was unremarkable. Autoimmune serologies, including ANCAs (MPO and PR3), were negative. There was no clinical, laboratory, or imaging evidence indicative of lymphoma, solid malignancy, or systemic autoimmune disease.

To assess for clonal or myeloproliferative variants of HES, molecular testing for the FIP1L1–PDGFRA fusion gene was negative. No hematologic abnormalities beyond eosinophilia were identified, and there were no features suggestive of myeloproliferative neoplasm. In the absence of clinical suspicion for clonal hematologic disease, bone marrow examination was not pursued.

With respect to organ involvement, although transthoracic echocardiography demonstrated impaired left ventricular diastolic function and chest CT revealed small bilateral pulmonary nodules, these findings were clinically stable and lacked supportive evidence for active eosinophil-mediated cardiopulmonary infiltration, such as elevated cardiac biomarkers, endomyocardial fibrosis, thrombus formation, or progressive pulmonary changes. Therefore, definitive cardiopulmonary organ damage attributable to eosinophilic disease could not be established. In contrast, cutaneous involvement and electrophysiologically confirmed peripheral neuropathy demonstrated a clear temporal association with marked eosinophilia and subsequent therapeutic response to eosinophil-targeted treatment, fulfilling diagnostic criteria for hypereosinophilic syndrome. The structured diagnostic evaluation is summarized in Table 2.

**Table 2:** Structured diagnostic work-up for hypereosinophilia.

**Category**	**Investigation**	**Result**	**Interpretation**
Baseline finding	Absolute eosinophil count	3.8 × 10^9^/L (peak)	Confirms hypereosinophilia
Parasitic infection	Stool O&P	Negative	Reactive cause unlikely
Helminth serology	Strongyloides IgG	Negative	Excluded
Drug-induced	Medication review	No new trigger	Unlikely
Allergic disease	Asthma history	Absent	EGPA less likely
ANCA	MPO, PR3	Negative	Against EGPA
Malignancy	CT chest/abdomen	No mass	No evidence
Myeloid HES	FIP1L1–PDGFRA	Negative	Exclusion of clonal PDGFRA
Serum tryptase	(value)	Normal	Against the myeloproliferative variant
Vitamin B12	(value)	Normal	Against the myeloid variant
T-cell clonality	Flow cytometry	No aberrant T cells	Lymphocytic variant unlikely
Bone marrow	Not performed	—	No hematologic suspicion

Based on persistent marked eosinophilia, evidence of eosinophil-associated organ dysfunction (skin and peripheral nerves), and the exclusion of secondary and clonal etiologies, the condition was classified as idiopathic hypereosinophilic syndrome with cutaneous involvement and peripheral neuropathy. Key differential features among HES, EGPA, and atopic dermatitis are summarized in Table 3.

**Table 3:** Key differential features among HES, EGPA, and atopic dermatitis.

**Feature**	**HES**	**EGPA**	**Atopic Dermatitis**
Asthma	Usually absent	Common	May coexist
ANCA	Negative	30–40% positive	Negative
Eosinophils	Markedly elevated (>1.5 × 10^9^/L)	Elevated	Mild–moderate
IgE	Often elevated	Elevated	Elevated
Skin biopsy	Eosinophilic infiltration, no vasculitis	Vasculitis ± eosinophils	Spongiotic dermatitis
Neuropathy pattern	Symmetric axonal polyneuropathy	Mononeuritis multiplex	Rare
IENFD	Normal (if large fiber involvement)	May be reduced	Normal
Response to JAK inhibitor	Improves pruritus	Not standard	Improves dermatitis
Response to anti-IL-5	Effective	Effective	Not indicated

Treatment was subsequently adjusted to mepolizumab at a dose of 300 mg administered subcutaneously every 4 weeks, in accordance with the approved regimen for hypereosinophilic syndrome. One month after the initiation of anti-IL-5 therapy, the patient reported marked improvement in distal numbness and neuropathic pain, with the restoration of a stable gait. Clinician reassessment demonstrated stabilization of sensory deficits without progression and sustained control of cutaneous manifestations. The patient demonstrated good adherence to therapy and tolerated treatment well, with no thromboembolic events or other serious adverse effects observed during follow-up.

Because the patient had previously received care at multiple institutions over a 10-year period and complete historical records were unavailable, detailed longitudinal laboratory data and exact dates of prior interventions could not be fully reconstructed. Only limited historical absolute eosinophil count (AEC) and IgE data were accessible. The clinical timeline presented in this report, therefore, reflects the most reliable confirmed information from the current hospitalization and available prior documentation.

## Discussion

The 10-year course of severe pruritus in this patient, which ultimately evolved into prominent peripheral sensory neuropathy, highlights a critical diagnostic pitfall in eosinophilic disorders, in which cutaneous manifestations dominate the clinical presentation while neurological involvement remains concealed. Early in the disease course, the coexistence of refractory pruritic rash and marked eosinophilia poses substantial diagnostic challenges in differentiating AD, HES, and ANCA-negative EGPA. In this case, the persistent, treatment-resistant skin lesions shared several features with AD, reflecting a well-recognized but complex clinical scenario: AD may be accompanied by eosinophilia; however, the emergence of multisystem involvement—particularly neurological manifestations—should prompt clinicians to move beyond a purely dermatologic diagnosis and consider systemic eosinophilic disorders. As reported in cases described by Merlotto and Long, erythroderma and pruritic eruptions are common cutaneous manifestations of HES and frequently contribute to delayed diagnosis (3, 8), a pattern that closely mirrors the early diagnostic dilemma encountered in the present case.

Distinguishing HES from ANCA-negative EGPA was central to the revised diagnosis in this case. When asthma is absent, and ANCA testing is negative, the clinical manifestations of these two entities overlap substantially, making differentiation particularly challenging. A critical distinguishing feature lies in the pattern of neuropathy and the underlying pathological mechanisms. Takeuchi et al. identified a key discriminating point, demonstrating that HES-associated neuropathy more commonly presents as a symmetric, length-dependent polyneuropathy, whereas EGPA typically manifests as mononeuritis multiplex (5). In the present case, both the electrophysiological findings and clinical course revealed progressive, diffuse, and symmetric sensorimotor nerve involvement, which is characteristic of HES-related neuropathy and contrasts with the focal, asymmetric pattern usually observed in EGPA.

From a pathological perspective, studies by Nishi *et al.* have further elucidated the mechanistic differences between the two conditions. In ANCA-negative EGPA, eosinophil-mediated vascular occlusion and direct cytotoxic effects are more prominent contributors to neural injury, often accompanied by inflammatory cell infiltration, whereas histopathological evidence of vasculitis—such as fibrinoid necrosis—is exceedingly rare in HES (9). In our patient, skin biopsy demonstrated scattered eosinophilic infiltration without definitive features of necrotizing vasculitis, further supporting an eosinophil-mediated toxic injury mechanism consistent with HES rather than EGPA.

This case illustrates a highly instructive clinical phenomenon. For nearly a decade, the patient was dominated by severe pruritus, during which progressively evolving neurological symptoms—including numbness, hypoesthesia, and even balance disturbance—were completely obscured and remained unrecognized. Following effective antipruritic therapy with upadacitinib, these neurological manifestations became more apparent and were more clearly reported by the patient. The temporal sequence observed in this case raises the possibility of sensory masking. It is conceivable that intense and persistent pruritic inputs may have functionally overshadowed concurrent but less prominent neuropathic sensory disturbances, thereby delaying their recognition. From a neurophysiological perspective, high-intensity pruritic signals may compete with other sensory inputs within shared central processing pathways, including spinal dorsal horn circuits and higher integrative centers, as suggested by models of sensory gating and competitive interaction between itch and pain pathways (10, 11). At the same time, alternative explanations should be acknowledged. Peripheral neuropathy may have progressed independently of itch severity, and subtle neurological symptoms may have been present earlier but underreported. The retrospective nature of symptom assessment also introduces the possibility of reporting or recall bias. In addition, medication adjustments may influence symptom perception. Therefore, while the temporal association supports a biologically plausible hypothesis of sensory masking, causality cannot be definitively established in this single case. This observation nonetheless carries important clinical implications. In patients presenting with chronic, refractory, and systemic pruritus—particularly when accompanied by marked eosinophilia—early and proactive evaluation of the peripheral nervous system should be considered, even in the absence of overt neurological complaints. NCSs and EMG may help detect subclinical axonal neuropathy and potentially reduce diagnostic delay.

Previous studies have demonstrated that peripheral neuropathy associated with HES most commonly presents as a sensory-predominant, axonal polyneuropathy with distal predominance, while true necrotizing vasculitis is relatively uncommon. Electrophysiological examinations typically reveal reduced sensory nerve amplitudes and slowed conduction velocities, whereas motor nerve function is relatively preserved (5, 12). In the present case, nerve conduction studies showed widespread sensory nerve involvement, with particularly prominent impairment of distal sensory nerves in the lower extremities, which is consistent with the characteristic electrophysiological pattern of HES-related peripheral neuropathy. In contrast, PGP9.5 immunostaining in skin biopsy specimens is primarily used to assess IENFD and reflects the structural integrity of small-diameter unmyelinated or thinly myelinated sensory fibers (C fibers and Aδ fibers). In the present case, the IENFD was within the age-adjusted reference range, indicating preservation of small intraepidermal nerve fibers. However, this technique does not evaluate large-diameter myelinated sensory fibers nor does it capture pathological changes at the level of nerve trunks or fascicles. Therefore, normal IENFD does not exclude peripheral neuropathy, particularly when large myelinated sensory fibers are predominantly affected, as demonstrated by electrophysiological abnormalities (13). This apparent dissociation does not represent a contradiction between diagnostic modalities; rather, it reflects the selective vulnerability of specific neural compartments and fiber types in HES-related neuropathy. At the level of lesion distribution, HES-related peripheral neuropathy tends to preferentially involve proximal neural structures, including nerve trunks, fascicles, and the endoneurium, rather than distal intraepidermal nerve endings (5). With respect to fiber type involvement, axonal damage to large-diameter myelinated sensory fibers is most prominent, while small fibers and motor fibers are relatively spared. This selective pattern may be attributable to eosinophilic infiltration, release of cytotoxic granule proteins, and chronic ischemia secondary to endoneurial microcirculatory disturbance rather than a classic necrotizing vasculitic process.

Initial treatment with upadacitinib, a Janus kinase (JAK) inhibitor, effectively controlled cutaneous inflammation and pruritus by broadly suppressing Th2 cytokine signaling; however, its capacity to reverse established axonal injury appeared limited. In contrast, after switching to mepolizumab, an anti-interleukin-5 monoclonal antibody, the patient’s neurological symptoms improved rapidly. This clinical response provides direct evidence that eosinophils serve as the central effector cells mediating neural injury in this context. By selectively blocking IL-5, mepolizumab induces profound depletion of eosinophils in both peripheral blood and tissues, thereby eliminating ongoing eosinophil-driven cytotoxicity and creating a permissive, non-inflammatory microenvironment for axonal repair. Compared with broad-spectrum immunosuppressive agents that primarily modulate downstream inflammatory pathways, this approach is more targeted and mechanistically fundamental. Our findings further support the IL-5 pathway as a rational and effective therapeutic target for HES-associated peripheral neuropathy (14).

Several limitations of this case report should be acknowledged. First, a repeat electrophysiological assessment was not performed following anti-interleukin-5 therapy. Although the patient reported marked symptomatic improvement, objective follow-up nerve conduction studies were unavailable; therefore, electrophysiological recovery or stabilization could not be formally documented. Second, structured neurological outcome measures were not prospectively applied, and pruritus severity was retrospectively quantified, which may introduce recall bias. Third, longitudinal laboratory data were limited because the patient had received care at multiple institutions over a prolonged disease course. Finally, as a single case report, causal inferences regarding the proposed sensory masking phenomenon and treatment-related neuroprotection cannot be definitively established. These limitations should be considered when interpreting the findings.

In summary, this case report of HES with a 10-year delay in diagnosis illustrates that eosinophilic disorders presenting with prominent refractory pruritus may conceal the underlying neurological involvement for a prolonged period. Accurate diagnosis requires a high index of suspicion in patients with chronic pruritus accompanied by marked eosinophilia, with clinicians moving beyond cutaneous findings to perform a comprehensive systemic evaluation. Careful characterization of the neuropathy pattern (symmetric polyneuropathy vs. mononeuritis multiplex), together with ANCA status, asthma history, and histopathological findings, is pivotal for distinguishing HES from EGPA, as refractory pruritus may conceal neurological involvement and delay the recognition of neuropathy; therefore, early electrophysiological testing is essential. Finally, directly targeting eosinophils—such as with anti–interleukin-5 therapy—represents an effective therapeutic option for selected patients with HES. This case underscores that, in clinical practice, “pruritus” may not be merely a dermatologic symptom but a warning signal of a systemic disorder that warrants deeper investigation.

## Patient perspective

The patient reported that severe pruritus had dominated his daily life for many years and significantly impaired his sleep and quality of life. After initiating upadacitinib, he experienced substantial relief from itching and improved sleep, which allowed him to recognize persistent numbness and imbalance in his lower limbs. Following treatment with mepolizumab, he noted meaningful improvements in walking stability and sensory discomfort. He expressed relief at finally receiving a clear diagnosis and an effective treatment plan after years of uncertainty.

### Reporting guideline

This case report has been prepared in accordance with the CARE guidelines.

## Data Availability

The original contributions presented in the study are included in the article/Supplementary material, further inquiries can be directed to the corresponding author.

## References

[1] Klion AD. Approach to the patient with suspected hypereosinophilic syndrome. Hematology Am Soc Hematol Educ Program. (2022) 2022:47–54. doi: 10.1182/hematology.2022000367, 36485140 PMC9821533

[2] Valent P Klion AD Horny HP Roufosse F Gotlib J Weller PF . Contemporary consensus proposal on criteria and classification of eosinophilic disorders and related syndromes. J Allergy Clin Immunol. (2012) 130:607–612.e9. doi: 10.1016/j.jaci.2012.02.019, 22460074 PMC4091810

[3] Long C Scott JL Flamm A. The dermatologic and histologic spectrum of hypereosinophilic syndrome. JAAD Case Rep. (2023) 39:21–5. doi: 10.1016/j.jdcr.2023.06.020, 37560139 PMC10407024

[4] Fourzali K Yosipovitch G Maderal A. An approach to hypereosinophilic syndrome presenting with cutaneous features. Dermatitis. (2022) 33:387–95. doi: 10.1097/der.0000000000000803 36399530

[5] Takeuchi H Kawamura K Kawasaki T Oka N. Distinct features of hypereosinophilic syndrome with neuropathy from eosinophilic granulomatosis with polyangiitis. Front Neurol. (2022) 13:1057767. Published 2022 Nov 15. doi: 10.3389/fneur.2022.1057767, 36457867 PMC9705778

[6] Fijolek J Radzikowska E. Eosinophilic granulomatosis with polyangiitis - advances in pathogenesis, diagnosis, and treatment. Front Med (Lausanne). (2023) 10:1145257. doi: 10.3389/fmed.2023.1145257, 37215720 PMC10193253

[7] Koike H Nishi R Ohyama K Morozumi S Kawagashira Y Furukawa S . ANCA-associated Vasculitic neuropathies: a review. Neurol Ther. (2022) 11:21–38. doi: 10.1007/s40120-021-00315-7, 35044596 PMC8857368

[8] Merlotto MR Cantadori LO Sakabe D Miot HA. Case for diagnosis. Erythroderma as manifestation of hypereosinophilic syndrome. An Bras Dermatol. (2018) 93:451–3. doi: 10.1590/abd1806-4841.20187419, 29924226 PMC6001083

[9] Nishi R Koike H Ohyama K Fukami Y Ikeda S Kawagashira Y . Differential clinicopathologic features of EGPA-associated neuropathy with and without ANCA. Neurology. (2020) 94:e1726–37. doi: 10.1212/WNL.0000000000009309, 32217776

[10] Chen XJ Sun YG. Central circuit mechanisms of itch. Nat Commun. (2020) 11:3052. doi: 10.1038/s41467-020-16859-5, 32546780 PMC7297978

[11] Braz J Solorzano C Wang X Basbaum AI. Transmitting pain and itch messages: a contemporary view of the spinal cord circuits that generate gate control. Neuron. (2014) 82:522–36. doi: 10.1016/j.neuron.2014.01.018, 24811377 PMC4492533

[12] Ogbogu PU Bochner BS Butterfield JH Gleich GJ Huss-Marp J Kahn JE . Hypereosinophilic syndrome: a multicenter, retrospective analysis of clinical characteristics and response to therapy. J Allergy Clin Immunol. (2009) 124:1319–25.e3. doi: 10.1016/j.jaci.2009.09.022, 19910029 PMC2829669

[13] Lauria G Hsieh ST Johansson O Kennedy WR Leger JM Mellgren SI . European Federation of Neurological Societies/peripheral nerve society guideline on the use of skin biopsy in the diagnosis of small fiber neuropathy. Report of a joint task force of the European Federation of Neurological Societies and the peripheral nerve society. Eur J Neurol. (2010) 17:903–e49. doi: 10.1111/j.1468-1331.2010.03023.x, 20642627

[14] Roufosse F Kahn JE Rothenberg ME Wardlaw AJ Klion AD Kirby SY . Efficacy and safety of mepolizumab in hypereosinophilic syndrome: a phase III, randomized, placebo-controlled trial. J Allergy Clin Immunol. (2020) 146:1397–405. doi: 10.1016/j.jaci.2020.08.037, 32956756 PMC9579892

