# Supplementary material for: Case Report: Hypereosinophilic syndrome misdiagnosed as atopic dermatitis due to refractory pruritic rash masking peripheral neuropathy
Source: Front Med (Lausanne). 2026 Mar 25;13:1794649. doi: 10.3389/fmed.2026.1794649 (PMC13056620; doi:10.3389/fmed.2026.1794649)
Supplement: Supplementary file 1 [file Supplementary_file_1.docx]

**Supplementary Table S1. Detailed motor nerve conduction study results**

| **Nerve** | **Side** | **Distal Latency (ms)** | **CMAP Amplitude (mV)** | **Conduction Velocity (m/s)** | **Segment** |
| --- | --- | --- | --- | --- | --- |
| Median | Right | 3.2 | 7.1 | 57.1 | Wrist–Elbow |
| Median | Left | 3.4 | 6.8 | 56.4 | Wrist–Elbow |
| Ulnar | Right | 2.5 | 6.0 | 50.5 | Wrist–Below elbow |
| Ulnar | Right | 6.5 | 5.3 | 50.9 | Below–Above elbow |
| Ulnar | Left | 2.8 | 2.0 | 51.9 | Wrist–Below elbow |
| Ulnar | Left | 6.4 | 1.3 | 35.5 | Below–Above elbow |
| Peroneal | Right | 4.2 | 1.6 | 43.8 | Ankle–Below fibular head |
| Tibial | Right | 4.3 | 7.0 | 48.0 | Ankle–Popliteal fossa |

**Supplementary Table S2. Detailed sensory nerve conduction study results**

| **Nerve** | **Side** | **Peak Latency (ms)** | **SNAP Amplitude (µV)** | **Conduction Velocity (m/s)** | **Result** |
| --- | --- | --- | --- | --- | --- |
| Median | Right | 2.3 | 9.8 | 40.6 | Reduced amplitude |
| Median | Left | 2.3 | 6.7 | 44.2 | Reduced amplitude |
| Ulnar | Right | NR | NR | NR | Absent |
| Ulnar | Left | NR | NR | NR | Absent |
| Radial | Right | 2.0 | 5.3 | 45.9 | Reduced amplitude |
| Radial | Left | 2.1 | 4.6 | 49.1 | Reduced amplitude |
| Sural | Bilateral | NR | NR | NR | Absent |
| Superficial peroneal | Bilateral | NR | NR | NR | Absent |

**Abbreviation:** NR, not recordable.

**Supplementary Table S3. F-wave and needle electromyography findings**

| **Examination** | **Nerve / Muscle Group** | **Findings** | **Interpretation** |
| --- | --- | --- | --- |
| F-wave | Median (Right) | Present (25.6 ms) | Normal |
|  | Tibial (Right) | Present (48.2 ms) | Normal |
| Needle EMG | Right upper limb | No spontaneous activity | Normal |
|  | Right lower limb | No fibrillation potentials or PSWs | Normal |
|  | Motor unit potentials | Normal morphology | No chronic neurogenic change |
